# Experimental priming of encephalitogenic Th1/Th17 cells requires pertussis toxin-driven IL-1β production by myeloid cells

**DOI:** 10.1038/ncomms11541

**Published:** 2016-05-18

**Authors:** Francesca Ronchi, Camilla Basso, Silvia Preite, Andrea Reboldi, Dirk Baumjohann, Luana Perlini, Antonio Lanzavecchia, Federica Sallusto

**Affiliations:** 1 Cellular Immunology Laboratory and Immune Regulation Laboratory, Institute for Research in Biomedicine, Università della Svizzera italiana, Via Vincenzo Vela 6, CH-6500 Bellinzona, Switzerland; 2 Institute of Microbiology, D-BIOL, ETH Zurich, Vladimir-Prelog-Weg 4, CH-8093 Zurich, Switzerland

## Abstract

CD4^+^ Th17 are heterogeneous in terms of cytokine production and capacity to initiate autoimmune diseases, such as experimental autoimmune encephalomyelitis (EAE). Here we demonstrate that experimental priming of encephalitogenic Th cells expressing RORγt and T-bet and producing IL-17A, IFN-γ and GM-CSF but not IL-10 (Th1/Th17), is dependent on the presence of pertussis toxin (PTX) at the time of immunization. PTX induces early production of IL-1β by CD11b^+^CCR2^+^Gr1^+^ myeloid cells, which are rapidly recruited to antigen-draining lymph nodes. PTX-induced generation of Th1/Th17 cells is impaired in IL-1β- and ASC-deficient mice and in mice in which myeloid cells are depleted or fail to migrate to lymph nodes and requires expression of IL-1R1 and MyD88 on both T cells and non-T cells. Collectively, these data shed light on the enigmatic function of PTX in EAE induction and suggest that inflammatory monocytes and microbial infection can influence differentiation of pathogenic Th1/Th17 cells in autoimmune diseases through production of IL-1β.

## Abstract (web summary)

Pertussis toxin enhances the induction of autoreactive T cells in mouse models of autoimmunity. Here the authors show that stimulation of IL-1β production in myeloid cells by pertussis toxin is necessary to prime pathogenic Th1/Th17 cells in experimental autoimmune encephalopathy.

## Graphical abstract

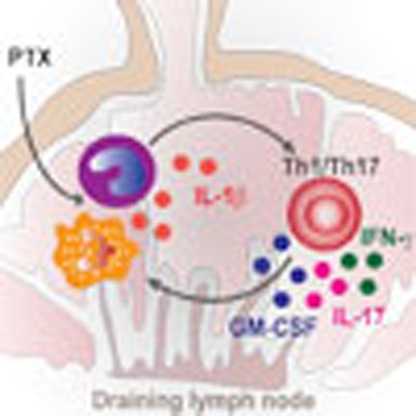

Experimental autoimmune encephalomyelitis (EAE) is a well-established mouse model of multiple sclerosis (MS), a debilitating inflammatory demyelinating disease of the human central nervous system (CNS). Early studies established that interleukin (IL)-17-producing CD4^+^ Th17 cells are required to induce EAE, as mice lacking RORγt, the Th17-specifying transcription factor, or IL-23, a Th17 growth and differentiation factor, are resistant to EAE induction^1^,^2^,^3^. However, further studies showed that not all Th17 are pathogenic. In particular, it has been demonstrated that Th17 cells primed *in vitro* in the presence of transforming growth factor (TGF)-β1 and IL-6 and producing IL-17 and IL-10 are non-pathogenic when transferred *in vivo* in a passive model of EAE^4^,^5^. In contrast, Th17 cells generated *in vitro* in the presence of IL-6, IL-23 and IL-1β or TGF-β3 and IL-6 and producing IL-17 together with interferon (IFN)-γ are pathogenic *in vivo*^5^,^6^. Furthermore, it has been shown that granulocyte–macrophage colony-stimulating factor (GM-CSF) production by Th17 cells, which is induced by IL-23 and RORγt, is required for initiation of CNS inflammation^7^,^8^,^9^. A better definition of the stimuli that induce the differentiation of IL-17^+^/IFN-γ^+^/GM-CSF^+^ T cells *in vivo* is essential to understand the initial events that may lead to autoimmunity.

To trigger EAE *in vivo*, myelin oligodendrocyte glycoprotein (MOG) or myelin proteolipid protein peptides are admixed with complete Freund's adjuvant (CFA) and injected subcutaneously into mice or rats^10^. The addition at the time of immunization of pertussis toxin (PTX)^11^, the major virulence factor of *Bordetella pertussis*, was empirically found to greatly improve the efficiency of EAE induction^12^,^13^ and to facilitate other murine autoimmune disease models, such as experimental autoimmune myocarditis, experimental autoimmune uveitis and glucose-6-phosphate isomerase (GPI)-induced arthritis^14^,^15^,^16^. However, in spite of the wide use, the mechanism by which PTX exerts its potent adjuvant effect on the immune response has remained elusive. Early studies indicated that PTX can increase permeability of blood vessels^17^,^18^, while more recently it was found that PTX can promote TLR4-dependent production of IL-1β and IL-6 by myeloid and stromal cells and entry of leukocytes in the CNS in the effector phase of EAE^19^.

In this study, we asked whether and how PTX can affect the early events of T-cell differentiation. We report that PTX promotes the priming of CD4^+^ T cells producing IL-17A, IFN-γ and GM-CSF, but not IL-10 (also defined as Th1/Th17), by inducing the rapid recruitment of neutrophils and inflammatory monocytes in the antigen-draining lymph nodes and production of IL-1β, which is required for the expansion and differentiation of encephalitogenic Th1/Th17 cells *in vivo*.

## Results

### PTX promotes development of encephalitogenic Th1/Th17 cells

**Figure 1:**
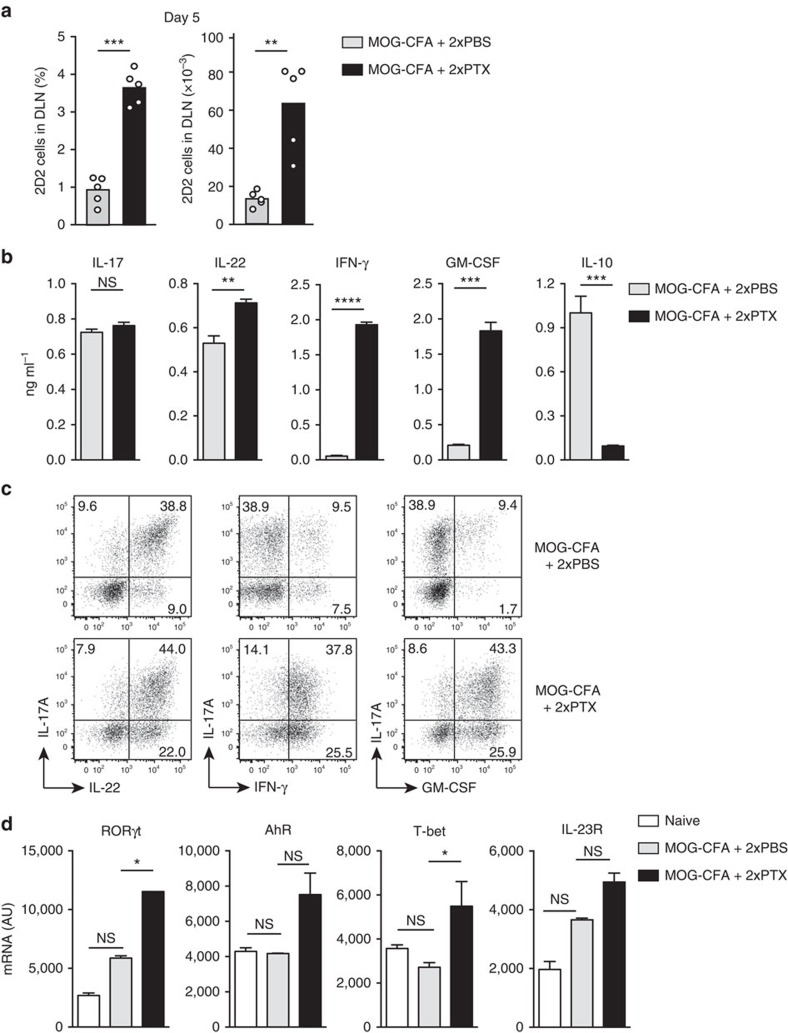
PTX induces encephalitogenic Th1/17 cells. (**a**) Mean percentage and absolute number of CD90.1^+^ CD4^+^ 2D2 TCR-transgenic T cells measured by flow cytometry in the draining lymph nodes (DLN) of CD90.2^+^ C57BL/6 mice on day 5 after immunization with MOG-CFA and injection of PBS or PTX on day 0 and day 2. Each symbol represents an individual mouse (*n*=5). Data are representative of more than five independent experiments, with at least 3 mice per group. (**b**) IL-17A, IL-22, IFN-γ, GM-CSF and IL-10 protein abundance in culture supernatants of flow cytometry-sorted CD90.1^+^ CD4^+^ 2D2 T cells isolated from DLN of immunized mice and restimulated *in vitro* with bone marrow-derived LPS-matured DCs pulsed with 20 μg ml^−1^ MOG_35–55_. Cytokines were assessed by ELISA after 72 h of culture. Data are mean+s.e.m. (*n*=3) and are representative of two independent experiments with 3–4 mice per group. (**c**) Representative flow cytometry analysis of cytokine staining of CD90.1^+^ CD4^+^ 2D2 T cells isolated from DLN of mice on day 5 after immunization and restimulated *in vitro* for 5 h with phorbol 12-myristate 13-acetate (PMA) and ionomycin in the presence of brefeldin A (BFA) for the last 3 h. Data are representative of more than five independent experiments with at least 3 mice per group. (**d**) T-bet, RORγt, AhR and IL-23R mRNA abundance in 2D2 T cells. CD90.1^+^ CD4^+^ 2D2 T cells were isolated from DLN of mice on day 5 after immunization and analysed by reverse transcription–PCR. Naïve CD4^+^ T cells were included as control. AU, arbitrary units. Data are mean+s.e.m. (*n*=3) and are representative of two independent experiments with 3–4 mice per group. *P<0.05, **P<0.01, ***P<0.001, ****P<0.0001, as determined by nonparametric unpaired Mann–Whitney test. NS, not significant.

To investigate the role of PTX in the early events of EAE induction, we adoptively transferred CD90.1^+^ MOG-specific naive 2D2 TCR-transgenic T cells into CD90.2^+^ C57BL/6 wild-type (WT) mice and followed their fate upon subcutaneous (s.c.) immunization with MOG peptide (MOG_35–55_) in CFA (MOG-CFA). PTX (200 ng per mouse) or PBS was injected intravenously (i.v.) on days 0 and 2. As shown in Fig. 1a, on day 5 after immunization, the frequency and absolute numbers of 2D2 T cells in the draining lymph nodes were significantly higher in PTX-treated mice as compared with PBS-treated control mice. 2D2 T cells were also found in higher numbers in the spleen and the brain of PTX-treated mice, peaking on day 7 (Supplementary Fig. 1a). 2D2 T cells from PTX-treated mice were larger and enriched in lipid rafts and were able to proliferate *in vitro* at lower concentrations of MOG as compared with 2D2 T cells from lymph nodes of PBS-treated mice (Supplementary Fig. 1b,c). Remarkably, although following restimulation *in vitro* with MOG, 2D2 T cells from PTX- and PBS-treated mice produced IL-17 at comparable levels, only 2D2 T cells from PTX-treated mice produced high levels of IFN-γ and GM-CSF and only 2D2 T cells from PBS-treated produced IL-10 (Fig. 1b). 2D2 T cells from PTX-treated mice produced also IL-22 in significantly higher amounts compared with cells from PBS-treated mice. These data were confirmed by intracellular cytokine staining that showed that a large proportion of 2D2 T cells from PTX-treated mice produced simultaneously IL-17, IFN-γ and GM-CSF (Fig. 1c). Consistent with the cytokine profile, T-bet and RORγt mRNAs were more abundantly expressed by 2D2 T cells from PTX-treated mice, whereas mRNAs for the arylhydrocarbon receptor (AhR) and IL-23R were expressed at comparable levels (Fig. 1d). Injection of PTX in MOG-CFA-immunized WT mice (with no adoptive transfer of 2D2 T cells) resulted in a higher proportion of endogenous CD4^+^ T cells expressing CD40L and producing IL-17, IFN-γ and GM-CSF upon restimulation *in vitro* with MOG (Supplementary Fig. 2a,b), indicating that the effect of PTX is not restricted to transgenic 2D2 T cells. As expected, PTX-treated, but not PBS-treated, mice developed EAE following immunization, with prominent infiltration of CD4^+^ T lymphocytes in the CNS (Supplementary Fig. 3a–c). Collectively, these data indicate that PTX potently synergizes with CFA to promote the early expansion and differentiation of highly responsive and encephalitogenic T cells that produce IL-17A, IFN-γ and GM-CSF and no IL-10 (hereafter defined as Th1/Th17).

### The synergistic effect of PTX requires enzymatic activity

To determine whether PTX could synergize with other adjuvants and in different experimental settings, we adoptively transferred CD4^+^ or CD8^+^ TCR transgenic T cells (2D2 T cells specific for MOG, OT-II and OT-I cells specific for ovalbumin, TCR7 cells specific for hen egg lysozyme) into congenic mice, which were then immunized with the relevant antigen together with CFA, LPS or bacterial extracts. In all cases, we observed that the enzymatically active PTX dramatically increased the proportion of T cells that produced three or more cytokines (IL-17A, IFN-γ, IL-22 and/or GM-CSF), also defined as multifunctional T cells (Supplementary Fig. 4a–c). In contrast, a non-toxic PTX mutant devoid of ADP-ribosylating activity^20^ failed to synergized with CFA (Supplementary Fig. 4d,e). We concluded that the synergistic effect of PTX in the induction of multifunctional T cells can be observed with different antigens and adjuvants and is dependent from the PTX enzymatic activity.

### PTX-induction of Th1/Th17 cells requires IL-1β but not IL-23

**Figure 2:**
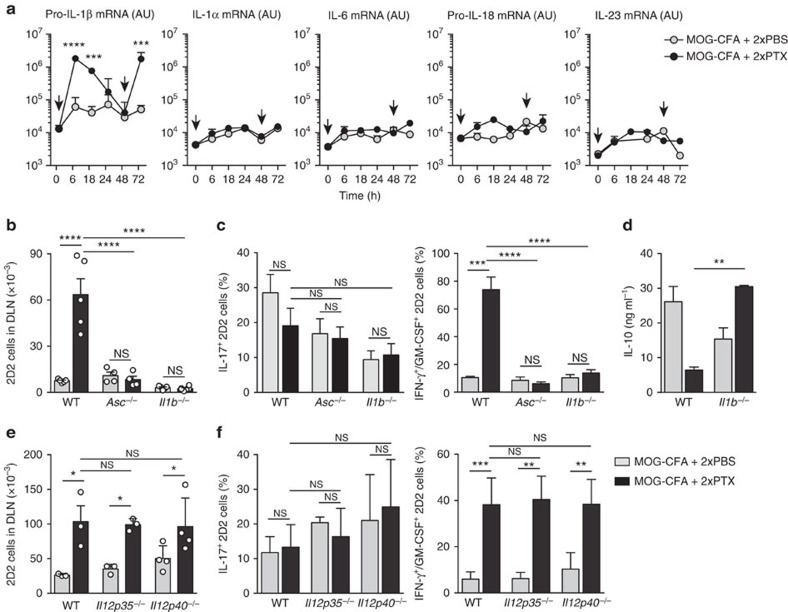
IL-1β is required for priming of Th1/17 cells. (**a**) Cytokine mRNA abundance measured by quantitative PCR in DLN at different time points after immunization with MOG-CFA and injection of PBS or PTX on day 0 and day 2 (arrows). Data are mean+s.e.m. (*n*=4) and are representative of two independent experiments with 3–4 mice per group. AU, arbitrary units. (**b**) Absolute number of CD90.1^+^ CD4^+^ 2D2 T cells measured by flow cytometry in DLN from WT, *Asc*^−/−^ and *Il1b*^−/−^ mice on day 5 after immunization. Each symbol represents an individual mouse. Data are mean+s.e.m. (*n*=4–6) and are representative of more than three independent experiments with at least 3 mice per group. (**c**,**d**) Percentage of IL-17^+^ and IFN-γ^+^/GM-CSF^+^ 2D2 T cells measured by intracellular cytokine staining (**c**) and concentration of IL-10 in culture supernatants measured by ELISA (**d**) of 2D2 T cells from DLN of WT or *Il1b*^−/−^ mice on day 5 after immunization. CD90.1^+^ CD4^+^ 2D2 T cells were sorted and stimulated *in vitro* for 5 h with PMA and ionomycin in the presence of BFA for the last 3 h (**c**) or for 72 h with LPS-matured MOG_35–55_-pulsed DCs (**d**). Data are mean+s.e.m. (*n*=3) and are representative of two independent experiments with at least 3 mice per group. (**e**) Absolute number of 2D2 T cells (CD90.1^+^ CD4^+^) in DLN of WT, *Il12p35*^−/−^ and *Il12p40*^−/−^ mice measured by flow cytometry on day 5 after immunization with MOG-CFA and injection of PBS or PTX on day 0 and day 2. Each symbol represents an individual mouse. Data are mean+s.e.m. (*n*=3–4) and are representative of two independent experiments with at least 3 mice per group. (**f**) Percentage of IL-17^+^ and IFN-γ^+^/GM-CSF^+^ 2D2 T cells measured by intracellular cytokine staining in DLN of WT, *Il12p35*^−/−^ or *Il12p40*^−/−^ mice on day 5 after immunization. Data are mean+s.e.m. (*n*=3–5) and are representative of three independent experiments with at least 3 mice per group. *P<0.05; **P<0.01, ***P<0.001, ****P<0.0001, as determined by nonparametric unpaired Mann–Whitney test. NS, not significant.

To investigate the mechanisms that lead to the induction of Th1/Th17 cells, we first analysed the cytokines induced in the draining lymph nodes at different time points after immunization with MOG-CFA and injection of PBS or PTX on day 0 and day 2. PBS injection did not induce upregulation of any of the cytokines analysed (Fig. 2a). In contrast, each PTX injection was followed by a rapid increase, up to 100-fold, of pro-IL-1β mRNA (Fig. 2a), whereas mRNAs for other cytokine genes, such as IL-1α, IL-6, pro-IL-18 and IL-23, were not significantly affected. Second, we transferred naïve CD90.1^+^ 2D2 T cells into CD90.2^+^ C57BL/6 mice lacking IL-1β, IL-12 or IL-23. Mice were immunized and T cells in the draining lymph nodes were analysed at day 5. When transferred into *Il1b*^–/–^ or *Asc*^–/–^ mice (both lacking mature IL-1β), 2D2 T cells expanded poorly and, although they acquired the capacity to produce IL-17, did not acquire the capacity to produce IFN-γ and GM-CSF and produced high amounts of IL-10 (Fig. 2b–d). As expected, when analysed at late time points, *Il1b*^–/–^ and *Asc*^–/–^ mice did not develop or developed a significantly milder disease compared with WT control mice (Supplementary Fig. 5a). In contrast, when transferred into *Il12p35*^−/−^ mice (that lack IL-12 and IL-35) or *Il12p40*^–/–^ mice (that lack IL-12 and IL-23), 2D2 T cells expanded and differentiated to Th1/Th17 cells as in WT mice (Fig. 2e,f). Differentiation of Th1/Th17 cells was also observed in IL-23R-deficient OT-II T cells transferred in WT mice immunized with OVA-CFA+PTX (Supplementary Fig. 5b). However, mice lacking IL-23 (*Il12p40*^–/–^ and *Il23p19*^–/–^) did not develop EAE (Supplementary Fig. 5c), consistent with previous results^21^,^22^ and supporting the notion that IL-23 is required for amplification and stabilization of the Th1/Th17 phenotype^23^.

Collectively, these observations imply that the key factor for the initial priming of encephalitogenic Th1/Th17 cells is IL-1β, which is acutely induced by PTX in the draining lymph nodes.

### PTX induces recruitment of IL-1β-producing myeloid cells

**Figure 3:**
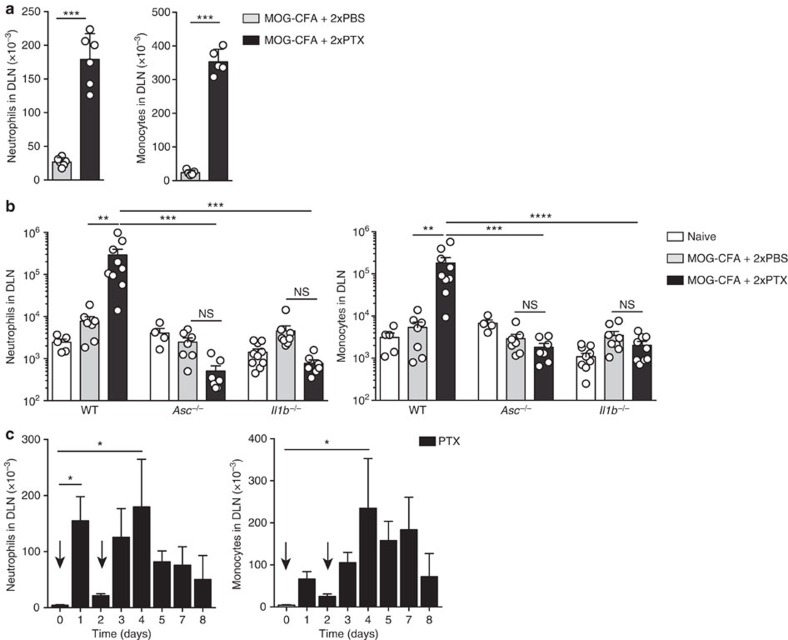
PTX induces recruitment of neutrophils and inflammatory monocytes in draining lymph nodes. (**a**) Absolute number of CD11b^+^ Gr1^hi^ Ly6C^int^ Ly6G^+^ neutrophils and CD11b^+^ Gr1^int^ Ly6C^hi^ Ly6G^−^ monocytes in DLN of mice 72 h after immunization with MOG-CFA and injection of PBS or PTX on day 0 and day 2. Each symbol represents an individual mouse. Data are mean+s.e.m. (*n*=6) and are representative of more than five independent experiments with at least 3 mice per group. (**b**) Absolute number of neutrophils and monocytes in DLN of WT, *Asc*^−/−^ and *Il1b*^−/−^ mice not immunized (naïve) or 72 h after immunization. Each symbol represents an individual mouse. Data are mean+s.e.m. (*n*=4–10) and are representative of more than three independent experiments with at least 3 mice per group. (**c**) Absolute number of neutrophils and monocytes in lymph nodes of mice at different time points after 1 or 2 injections of PTX alone on day 0 and 2 (arrows). Data are mean+s.e.m. (*n*=3) and are representative of more than three independent experiments with at least 3 mice per group. *P<0.05; **P<0.01; ***P<0.001, ****P<0.0001, as determined by nonparametric unpaired Mann–Whitney test. NS, not significant.

To further investigate the mechanisms that lead to PTX-dependent IL-1β production, we analysed the cellular composition of the draining lymph nodes of mice immunized with MOG-CFA and injected with PTX or PBS on day 0 and day 2. On day 5 after immunization, the absolute numbers of CD11b^+^ Gr1^hi^ Ly6C^int^ Ly6G^+^ neutrophils and CD11b^+^ Gr1^int^ Ly6C^hi^ Ly6G^–^ inflammatory monocytes in the draining lymph nodes dramatically increased in PTX-treated mice as compared with PBS-treated control mice (Fig. 3a). Several inflammatory chemokines, including CXCL2, CCL2 and CXCL10, were detected at high levels in the draining lymph nodes of immunized mice injected with PTX, but not with PBS (Supplementary Fig. 6a,b), providing a plausible mechanism for the recruitment of neutrophils and monocytes that express the cognate receptors CXCR1, CXCR2 and CCR2. Importantly, PTX injection in *Asc*^–/–^ and *Il1b*^–/–^ mice did not induce recruitment of myeloid cells in the draining lymph node and did not trigger chemokine production (Fig. 3b and Supplementary Fig. 6c). Interestingly, in WT mice, injection of PTX alone on day 0 and day 2 induced neutrophil and monocyte recruitment in lymph nodes (Fig. 3c). In particular, although the first injection of PTX led to a transient increase in the number of neutrophils and monocytes, the second injection resulted in a sustained increase that lasted for several days. Injection of PTX alone induced also recruitment of neutrophils and monocytes in the spleen and the brain (Supplementary Fig. 7a,b).

**Figure 4:**
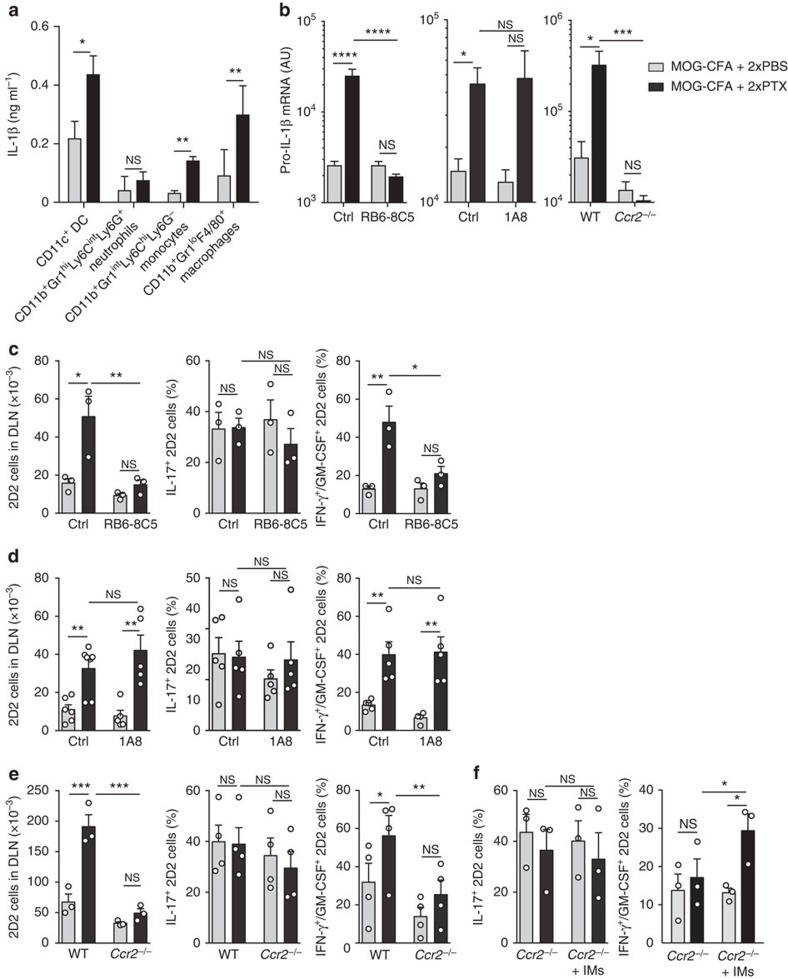
Myeloid cells in PTX-draining lymph nodes produce IL-1β. (**a**) Spontaneous production of IL-1β by different myeloid cells sorted from DLN 72 h after immunization with MOG-CFA and injection of PBS or PTX on day 0 and day 2. Sorted cells were cultured in medium for 16 h in the presence of ATP for the last 45 min and protein abundance was measured by ELISA. Data are mean+s.e.m. (*n*=4) and are representative of two independent experiments with at least 3 mice per group. (**b**) Pro-IL-1β mRNA abundance in DLN of WT mice injected with Gr1-depleting (RB6-8C5) antibody (left) or Ly6G-depleting (1A8) antibody (centre) or of *Ccr2*^−/−^ mice (right) 72 h after immunization. Isotype-matched antibodies were used as control (Ctrl). Data are mean+s.e.m. (*n*=6–9, *n*=5–6, *n*=3–4, respectively), and are representative of three independent experiments with at least 3 mice per group. AU, arbitrary units. (**c**–**e**) Absolute number of CD90.1^+^ CD4^+^ 2D2 T cells and percentage of IL-17^+^ and IFN-γ^+^/GM-CSF^+^ 2D2 T cells measured by surface and intracellular cytokine staining in DLN of WT mice injected with Gr1-depleting (RB6-8C5) antibody (**c**) or Ly6G-depleting (1A8) antibody (**d**) or of *Ccr2*^−/−^ mice (**e**) on day 5 after immunization. Isotype-matched antibodies were used as control (Ctrl). 2D2 T cells were directly stained or restimulated *in vitro* for 5 h with PMA and ionomycin, in the presence of BFA for the last 3 h and then stained for intracellular cytokines. Data are mean+s.e.m. (*n*=3, *n*=5–7, *n*=3, respectively) and are representative of more than three independent experiments with at least 3 mice per group. Each symbol represents an individual mouse. (**f**) Percentage of IL-17^+^ and IFN-γ^+^/GM-CSF^+^ 2D2 T cells in DLN of *Ccr2*^−/−^ mice or of *Ccr2*^−/−^ mice receiving 3 × 10^6^ WT CCR2^+^ bone marrow-derived inflammatory monocytes on day 0 and 2 (+IMs). Data are mean+s.e.m. (*n*=3) and are representative of three separate experiments with 3–4 mice per group. *P<0.05, **P<0.01, ***P<0.001, ****, as determined by nonparametric unpaired Mann–Whitney test. NS, not significant.

To identify which cells in the draining lymph node of immunized mice were responsible for IL-1β production in response to PTX, we used two different approaches. First, we isolated from immunized mice treated with PBS or PTX different cell types and measured their spontaneous production of IL-1β. As shown in Fig. 4a, CD11c^+^ dendritic cells (DCs), CD11b^+^ Gr1^int^ Ly6C^hi^ Ly6G^–^ inflammatory monocytes and F4/80^+^ macrophages isolated from PTX-treated mice, spontaneously released mature IL-1β when cultured in the absence of additional stimuli. Furthermore, both CD11c^+^ and CD11b^+^ cells produced mature IL-1β when exposed to PTX *in vitro* (Supplementary Fig. 8). Second, we depleted different cell populations *in vivo* and monitored expression of pro-IL-1β in the draining lymph nodes of immunized mice treated with PBS or PTX. A dramatic decrease in pro-IL-1β mRNA expression in draining lymph nodes was observed in mice treated with an anti-Gr1 antibody (RB6-8C5, depleting neutrophils and monocytes) and in *Ccr2*^–/–^ mice, in which recruitment of inflammatory myeloid cells is impaired, but not in mice treated with an anti-Ly6G antibody (1A8, depleting neutrophils; Fig. 4b). In contrast, depletion of F4/80^+^ macrophages (by clodronate liposome treatment, Supplementary Fig. 9) or CD11c^+^ cells (in bone marrow CD11c-DTR chimeric mice, Supplementary Fig. 10) did not result in significant changes in pro-IL-1β mRNA in immunized mice.

To address the potential role of Gr1^+^ cells, and in particular of CCR2^+^ monocytes, in the induction of Th1/Th17 cells, we analysed the phenotype of 2D2 T cells primed in mice that were depleted of these cells. In mice treated with the Gr1-depleting antibody and in *Ccr2*^–/–^ mice, but not in mice treated with the Ly6G-depleting antibody or depleted of F4/80^+^ or CD11c^+^ cells, 2D2 T cells did not expand in response to PTX and did not acquire the Th1/Th17 phenotype (Fig. 4c–e and Supplementary Figs 9 and 10). However, transfer of WT CCR2^+^ monocytes into *Ccr2*^–/–^ mice was sufficient to reconstitute the PTX-dependent differentiation of Th1/Th17 cells (Fig. 4f).

Collectively, these results indicate that PTX induces recruitment of IL-1β-producing CCR2^+^ myeloid cells required for priming of Th1/Th17 cells producing IL-17A, IFN-γ and GM-CSF.

### IL-1R1-signalling is required on T cells and non-T cells

**Figure 5:**
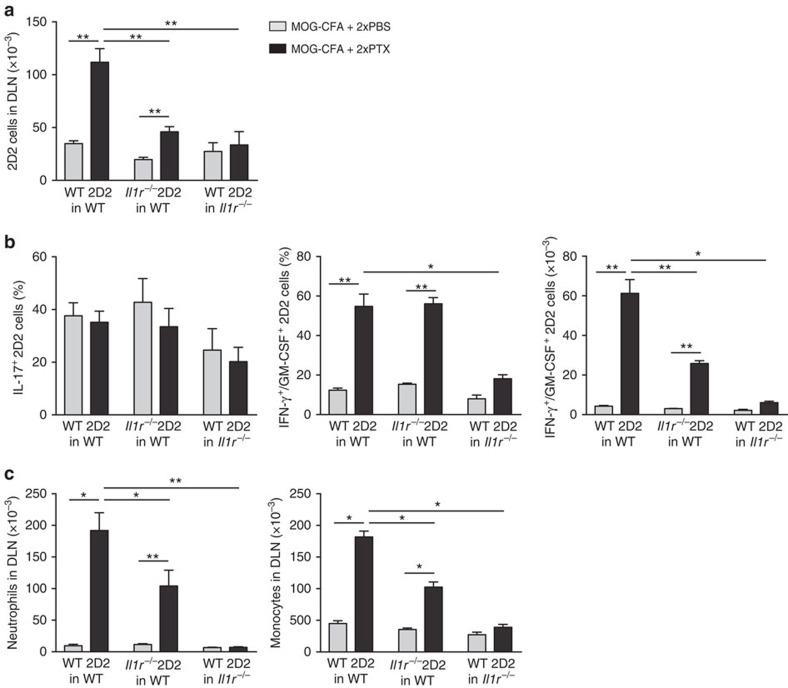
PTX effects require IL-1R signalling on T cells and non-T cells. (**a**) Absolute number of WT 2D2 T cells and *Il1r1*^*−/−*^ 2D2 T cells measured by flow cytometry in DLN of WT or *Il1r1*^*−/−*^ mice on day 5 after immunization with MOG-CFA and injection of PBS or PTX on day 0 and day 2. Data are mean+s.e.m. (*n*=4) and are representative of four independent experiments with 3–4 mice per group. (**b**) Percentage of IL-17^+^ and percentage and absolute numbers of IFN-γ^+^/GM-CSF^+^ WT or *Il1r1*^*−/−*^ 2D2 T cells in DLN of mice on day 5 after immunization. T cells were restimulated *in vitro* for 5 h with PMA and ionomycin in the presence of BFA for the last 3 h and then stained for intracellular cytokines. Data are mean+s.e.m. (*n*=4) and are representative of four independent experiments with 3–4 mice per group. (**c**) Absolute number of CD11b^+^ Gr1^hi^ Ly6C^int^ Ly6G^+^ neutrophils and CD11b^+^ Gr1^int^ Ly6C^hi^ Ly6G^−^ monocytes in DLN of WT or *Il1r1*^*−/−*^ mice on day 5 after immunization. Data are mean+s.e.m. (*n*=6) and are representative of three independent experiments with at least 3 mice per group. *P<0.05; **P<0.01, as determined by nonparametric unpaired Mann–Whitney test. When not indicated, differences were not statistically significant.

Having found a role for IL-1β in the recruitment of myeloid cells and in the differentiation of Th1/Th17 cells, we were interested to establish in which compartment IL-1β signalling was required. To this end, we crossed 2D2 transgenic mice with *Il1r1*^*–/–*^ mice and transferred *Il1r1*^*–/–*^ 2D2 T cells into WT hosts. We also transferred wt 2D2 T cells into *Il1r1*^*–/–*^ hosts. Mice were immunized with MOG-CFA and injected on days 0 and 2 with PBS or PTX. In WT recipient immunized with MOG-CFA alone, the expansion of WT and *Il1r1*^*–/–*^ 2D2 T cells was comparable (Fig. 5a), indicating that, in a CFA-driven response, IL-1β signalling is dispensable in T cells. In contrast, in WT recipient immunized with MOG-CFA and receiving PTX, expansion of *Il1r1*^*–/–*^ 2D2 T cells was significantly reduced (Fig. 5a). Although there was no significant reduction in the percentage of IL-17^+^ or IFN-γ^+^/GM-CSF^+^ cells, the absolute number of *Il1r1*^*–/–*^ 2D2 T cells that acquired the IFN-γ^+^/GM-CSF^+^ phenotype was significantly reduced (Fig. 5b). In these mice, recruitment of neutrophils and monocytes in the draining lymph node was also significantly impaired (Fig. 5c), suggesting a positive feedback mechanism induced by GM-CSF produced by Th1/Th17 cells in the recruitment or activation of myeloid cells in the draining lymph node^24^. In *Il1r1*^–/–^ hosts, PTX failed to induce expansion of adoptively transferred wt 2D2 T cells (Fig. 5a). In these mice, frequency and absolute number of WT 2D2 T cells that acquired the IFN-γ^+^/GM-CSF^+^ phenotype were significantly reduced (Fig. 5b). In addition, the PTX-induced recruitment of neutrophils and monocytes in the draining lymph nodes was abolished (Fig. 5c).

Finally, we analysed the response of WT 2D2 T cells transferred into mice deficient for MyD88, an adaptor molecule that is known to be required for IL-1R1 and Toll-like receptor (TLR) signalling^25^, and into *Tlr4*^–/–^, *Tlr2*^–/–^ or *Tlr*9^–/–^ mice. There was a marked reduction of 2D2 T-cell expansion and differentiation to Th1/Th17 cells and of recruitment of neutrophils and monocytes in immunized *Myd88*^–/–^ mice treated with PTX (Supplementary Fig. 11a–c). In contrast, although there was a slight reduction in the number of 2D2 T cells detected on day 5 in the draining lymph nodes, the differentiation to Th1/Th17 cells and the recruitment of neutrophils and monocytes were not affected in immunized *Tlr*^*–/–*^ mice (Supplementary Fig. 11d–f).

Collectively, these findings indicate that IL-1β can act directly on activated T cells to promote the expansion and differentiation of T cells producing IL-17, IFN-γ and GM-CSF. They also indicate that the IL-1R1-MyD88 signalling on host cells, most likely in the draining lymph nodes, is required for the recruitment of inflammatory myeloid cells and for the generation of encephalitogenic Th1/Th17 cells.

## Discussion

In this study, we define *in vivo* conditions that promote the rapid generation of Th1/Th17 cells producing IL-17, IFN-γ and GM-CSF, but not IL-10, and expressing RORγt and T-bet. The cytokine primarily involved in the differentiation of these highly inflammatory multifunctional T cells is IL-1β produced by CCR2^+^ myeloid cells that are recruited at early time points in the draining lymph nodes in a PTX-dependent manner. These findings reveal an *in vivo* mechanism for the generation of Th1/Th17 cells through microbial induction of IL-1β and provide an explanation for the enigmatic role of PTX in the enhancement of EAE.

PTX is used in most EAE models and in other models of autoimmune diseases, such as autoimmune myocarditis, autoimmune uveitis or GPI-induced arthritis^14^,^15^,^16^. In the EAE model, it has been shown that PTX facilitates leukocyte recruitment in the CNS by increasing permeability across the blood–brain barrier, an effect that is at least partly dependent on TLR4 (refs 19, 26). PTX injection was also shown to reduce frequency and suppressive activity of splenic Treg cells, whereas *in vitro* PTX-treated splenic cells, which produced IL-6 and other proinflammatory cytokines, overcame the inhibition of proliferation in co-cultures of Treg and CD4^+^CD25^–^ T effector cells^27^,^28^,^29^. *In vitro*, PTX induced generation of IL-17-producing T cells, an effect that was markedly inhibited by anti-TGF-β antibodies^29^. Our results show that *in vivo* PTX can act at early time points to promote the differentiation in draining lymph nodes of highly inflammatory T cells, producing IL-17, IFN-γ and GM-CSF, a finding that may explain its capacity to promote a broad variety of inflammatory diseases in different tissues. EAE may be dependent on the combined effects of cytokines produced by these multifunctional Th1/Th17 cells that can enhance recruitment and function of innate and adaptive immune cells in the brain and also activate microglia and impair the barrier function of brain endothelial cells^8^,^30^,^31^,^31^,^31^,^34^.

PTX is composed of five subunits (S1 to S5); the S1 subunit has the enzymatic activity that catalyses ADP-ribosylation of the α subunit of trimeric G proteins, leading to a variety of biological activities, whereas the S2–S5 subunits, which together form the B oligomer, are responsible for binding of the toxin to target cell receptors and internalization via receptor-mediated endocytosis^35^. Although it was shown that the oligomer B is sufficient to stimulate T-cell proliferation in the presence of APCs or fibroblasts^36^, our data demonstrate that the ADP-ribosyl transferase activity is required to induce myeloid cell recruitment and generation of Th1/Th17 cells. These data are consistent with those reported by Dumas *et al.*, showing that the ability of PTX to induce IL-1β transcription in peritoneal leukocytes and secretion of IL-1β and IL-6 in the peritoneal fluid depends on the integrity of its ADP-ribosyltransferase activity^19^.

It has been clearly established that Th17 cells come in different flavours and that not all Th17 cells are pathogenic^37^. The Th1/Th17 cells that are induced *in vivo* in the presence of PTX resemble the pathogenic Th17 cells induced by *in vitro* priming of naïve T cells in the presence of IL-23, IL-6 and IL-1β or TGF-β3 and IL-6 (refs 5, 6). In both cases, the cells express RORγt and T-bet and produce IL-17A and IFN-γ, but not IL-10. In addition, we have shown that the PTX-induced Th1/Th17 cells produce high amounts of GM-CSF, an essential cytokine to initiate neuroinflammation^7^,^8^,^9^. We have also found that while IL-1β was essential to induce Th1/Th17 in the antigen-draining lymph nodes, IL-23 or IL-12 were not required at this early time point, but IL-23 was required to trigger EAE. These data support the notion that different cytokines are involved in the early and late phases of EAE to generate and maintain encephalitogenic T cells^38^.

Previous studies have described a role of inflammatory monocytes in the effector phase of EAE. Mice that lack CCR2, a chemokine receptor that binds to monocyte chemoattractant protein 1 (MCP-1/CCL2) that is highly expressed in the inflamed CNS, have reduced number of inflammatory monocytes in the CNS, fail to develop EAE after active immunization and are resistant to induction of EAE by adoptive transfer of encephalitogenic T cells^39^,^40^. Moreover, studies in MCP-1-deficient mice have indicated an important role for the CCR2-MCP-1 axis in recruitment into the CNS of circulating monocytes and macrophage precursors, which are required for primed T cells to execute their effector functions in the tissue^41^. In this study, we have shown for the first time that the adjuvant effect of PTX is mediated through the CCR2-dependent early recruitment in lymph nodes of Gr1^+^ CCR2^+^ myeloid cells that produce high levels of IL-1β. It is possible that blocking monocyte extravasation in lymph nodes as well as in the CNS may result in a synergistic effect by limiting both generation and effector function of encephalitogenic T cells.

The important role of IL-1β in the induction of a subset of inflammatory Th1/Th17 cells and its involvement in autoimmune diseases such as MS was previously shown by several groups, including ours^42^,^43^,^44^,^45^,^46^. By linking PTX to IL-β production and induction of Th1/Th17 cells, our study provides a plausible explanation for the disease-inducing effect of PTX, in addition to the recently reported effects of PTX-induced IL-β in the upregulation of adhesion molecules on blood–brain capillaries that enhances the recruitment of inflammatory leukocyes into the brain^19^. The identification of endogenous or environmental factors that can affect human Th17 function in a way similar to those induced by PTX and described in this study may provide new insights into the pathogenic mechanisms of MS and other Th17-mediated autoimmune diseases.

## Methods

### Mice

C57BL/6J mice were obtained from Harlan (Italy). *Il1b*^−/−^ C57BL/6 mice^47^ were provided by Y. Iwakura and M. Bachmann. *Asc*^−/−^ C57BL/6 mice^48^ were provided by V. Dixit and J. Tschopp. *Il12p35*^−/−^, *Il23p19*^−/−^ and *Il12p40*^−/−^ C57BL/6 mice^49^ were provided by B. Becher. *Myd88*^−/−^, *Tlr4*^−/−^, *Tlr9*^−/−^ and *Tlr2*^−/−^ C57BL/6 mice^50^,^51^,^52^,^53^ were provided by S. Akira and M. Manz. *Il1r1*^*−/−*^ mice^54^ were provided by M. Kopf and backcrossed on C57BL/6 background for at least ten generation before use. *Ccr2*^−/−^ C57BL/6 mice^55^ were from Jackson Laboratory (004999). TCR7 TCR transgenic C57BL/6 mice^56^ were provided by A. O'Garra. OT-II and OT-I TCR-transgenic C57BL/6 mice^57^,^58^ were purchased from Jackson laboratory (004194 and 003831, respectively) and bred in our facility. 2D2 TCR-transgenic C57BL/6 mice^59^ (006912, Jackson Laboratory) were bred onto backgrounds of various CD90 alleles and crossed with *Il1r1*^*−/−*^ and *Myd88*^−/−^mice. Mice were maintained under specific pathogen-free conditions at the Institute for Research in Biomedicine Animal Facility. Mice were treated in accordance with guidelines of the Swiss Federal Veterinary Office and experiments were approved by the Dipartimento della Sanità e Socialità. Female mice were used between 5 and 10 weeks of age.

### EAE model and *in vivo* experiments

Mice were immunized s.c. on day 0 with 150 μg of MOG_35–55_ peptide (MEVVGWYRSPFSRVVHLYRNGK; Servei de Proteòmica, Pompeu Fabra University, Barcelona, Spain) emulsified in CFA (incomplete Freund's adjuvant plus 4 mg ml^−1^ of *Mycobacterium tuberculosis* H37RA, Difco). PTX (Sigma) was injected at 200 ng per injection per mouse in 200 μl saline i.v. on day 0 and day 2. Control mice received 200 μl of PBS only. In some experiments, MOG-CFA immunized mice were injected on day 0 and day 2 with a 9K/129G PTX mutant^20^ (provided by R. Rappuoli). Disease severity was assigned as described previouly^10^. For adoptive transfer, naïve CD90.1^+^ 2D2 T cells (0.3–1 × 10^5^ per mouse) were injected i.v. 16 h before immunization. In some adoptive transfer experiments, naive CD8^+^ OT-I cells, naive CD4^+^ TCR7 or naïve OT-II T cells (0.3–1 × 10^5^ per mouse) were injected i.v. 16 h before immunization. Then mice were immunized s.c. with the relevant antigen emulsified in CFA. MHC class I-binding peptide SIINFEKL (OVA amino acids 257–264, 15 μg per mouse) and OVA_323–339_ peptide (SQAVHAAHAEINEAGR 30 μg per mouse) were obtained from Servei de Proteòmica, Pompeu Fabra University, Barcelona, Spain. Hen egg lysozyme peptide (HEL_46–61_, 50 μg per mouse) was purchased from Anaspec. In other experiments, mice were immunized s.c. with antigen in combination with 15 μg per mouse of LPS (ultrapure *Escherichia coli* O111-BA LPS, Invivogen), 0.8 mg per mouse of *M. tuberculosis* (H37RA, Difco) or with *E. coli* or *Streptococcus pyogenes* (heat inactivated, pathogen extracts), as adjuvants. To deplete Gr1^+^ cells, α-Gr1 antibody (clone RB6-8C5 from BioXCell) or rat IgG2b isotype control antibody (clone LTF2 from BioXCell) was injected daily intraperitonally (i.p.) at a dose of 400 μg per mouse. To deplete Ly6G^+^ cells, α-Ly6G antibody (clone 1A8 from BioXCell) or rat IgG2a isotype control antibody (clone 2A3 from BioXCell) was injected daily i.p. at a dose of 400 μg per mouse. Frequency of neutrophils in lymph node was drastically reduced in RB6-8C5-treated mice compared with LTF2-treated mice (mean±s.e.m. (*n*=4): 12.1±3.9% versus 26.5±4.2% PBS, 16.7±4.0% versus 59.2±6.2% PTX) and in 1A8-treated mice compared with 2A3-treated mice (mean±s.e.m. (*n*=3): 1±0.6% versus 12.9±3.7% PBS, 4.5±2.6% versus 44.5±7.2% PTX). To deplete macrophages, clodronate-liposomes or control liposomes (1 mg per 200 μl) were administered i.p. daily, starting on day –2 before immunization^60^. CD11c-DTR bone marrow chimeras were generated through reconstitution of sublethally irradiated RAG1^*−/−*^ female mice with 2 × 10^6^ bone marrow cells from CD11c-GFP-DTR C57BL/6 donor female mice. Chimeras were used 2 months after successful reconstitution. Diphtheria toxin was administered daily i.p. for 5 days at a dose of 4 ng per g of body weight.

### Cell preparation and isolation from tissues

To isolate naïve T cells from the different mouse strains, cells from the spleen and lymph nodes were first enriched with anti-CD4 magnetic beads (L3T4, Miltenyi Biotec) and then sorted on a FACSAria Cell Sorter (BD Biosciences) to obtain cells with a CD4^+^CD8^−^CD25^−^CD44^low^CD62L^high^ phenotype. To study T cells primed *in vivo*, mice were killed on day 5, or as indicated, and CD90.1^+^ 2D2 T cells or endogenous CD90.1^−^CD90.2^+^ CD4^+^ T cells from lymphoid organs and the brain were first enriched with anti-CD90.1 or anti-CD4 beads (Miltenyi Biotech) and then further purified by FACS sorting. Dead cells were always excluded by staining with 7-aminoactinomycin D (7-AAD) (BioLegend). In preliminary experiments, day 5 was chosen as optimal time point for the analysis of T cells from draining lymph node (Supplementary Fig. 12). Myeloid cells from draining lymph nodes and lymphocytes from CNS were isolated after dissecting the organ and incubation of 45 min at 37 °C in a solution with 1 mg ml^−1^ of Collagenase D and 40 μg ml^−1^ of DNase I (Roche). Cells were then passed through a Nylon filter 40 μm and residues were smashed. After washing, cells from the CNS were separated on Percoll (GE Healthcare) gradient (70:37%) to obtain mononuclear cells. Bone marrow-derived DCs were generated according to a standard protocol^61^ using the supernatant of a GM-CSF-producing cell line^62^,^63^. *Il23r*^–/–^ splenocytes were provided by F. Powrie, and CD4^+^ naïve T cells were isolated as described above for adoptive transfer experiments. Inflammatory monocytes were isolated from bone marrow of WT animals by depletion of magnetically labelled T cells, B cells, NK cells, DCs, erythroid cells and granulocytes using the monocyte isolation kit (Miltenyi Biotech), as previously described^64^. In all preparations, Ly6G^hi^ neutrophils were less than 5% of CD11b^+^ isolated cells.

### Flow cytometry

For analysis of mouse cell phenotypes, the following monoclonal antibodies were used: α-CD3 (clone: 145-2C11), α-CD4 (RM4-5), α-CD8α (53–6.7), α-CD25 (PC61), α-CD44 (IM7), α-CD62L (MEL14), α-CD90.1 (OX-7), α-CD40L (MR-1), α-CD11c (HL3), α-CD11b (M1/70), α-Gr1 (RB6-8C5), α-Ly6C (HK1.4), α-Ly6G (1A8), α-F4/80 (BM8), α-IFN-γ (XMG1.2), α-IL-17A (TC11-18H10), α-GM-CSF (MP1-22E9; all from BioLegend) and α-IL-22 (AM22-3; provided by J.C. Renauld). For staining of lipid rafts, biotin-conjugated cholera toxin subunit B (Molecular Probes) was used together with fluorochrome-conjugated streptavidin (BioLegend). Dead cells were always excluded by staining with 7-AAD (BioLegend) or 4,6-diamidino-2-phenylindole (Sigma). All antibodies were used at 1 μg per 10^6^ cells. For intracellular cytokine staining, cells were stimulated for 5 h with phorbol 12-myristate 13-acetate (10^−7^ M; Sigma) and ionomycin (1 μg ml^−1^; Sigma), in the presence of brefeldin A (10 μg ml^−1^; Sigma) for the last 3 h of culture. Cells were fixed with 4% (wt/vol) paraformaldehyde and permeabilized with 0.5% (wt/vol) saponin (Sigma). Eight-colour staining was performed with the appropriate combinations of antibodies conjugated to fluorochromes. Samples were acquired on a FACSCanto II (BD Biosciences) and analysed with FlowJo software (TreeStar) and when necessary using the Boolean gating strategy^65^.

### *In vitro* experiments

7-AAD^–^ CD4^+^ CD90.1^+^ 2D2 T cells were sorted from draining lymph nodes on day 5 after immunization, labelled with carboxyfluorescein succinimidyl ester (CFSE) and cultured (2 × 10^4^ cells per well) in round-bottom 96-well plates with glutaraldehyde-fixed MOG_35–55_-pulsed, LPS-matured, DCs (1 × 10^4^ cells per well), as described previously^63^. In other experiments, 7-AAD^–^ CD4^+^ CD44^+^ T cells were sorted from draining lymph nodes on day 5 after immunization, labelled with CFSE and cultured (3 × 10^4^ cells per well) in round-bottom 96-well plates with irradiated MOG_35–55_-pulsed splenocytes from *Rag1*^–/–^ mice (5 × 10^4^ cells per well). T-cell proliferation was assessed by flow cytometry on the basis of CFSE dilution after 72 h of culture. Supernatants of the T-cell cultures were harvested and analysed by ELISA. DC stimulation was induced by overnight incubation with 0.5 μg ml^−1^ LPS (ultrapure *E. coli* O111-BA LPS, Invivogen). To induce IL-1β secretion, 0.1 mg ml^−1^ ATP-γ-S (Sigma) was added in the cell culture medium for the last 30–45 min of incubation. Supernatants of the culture was then harvested and analysed by ELISA.

### Quantitative real-time PCR

RNA was prepared from transferred 2D2 T cells or from total draining lymph node cells using TRIzol LS reagent (Invitrogen) according to the manufacturer's instructions. Draining lymph nodes were dissected, cut into pieces and incubated for 45 min at 37 °C with 1 mg/ml of Collagenase D and 40 μg/ml of DNase I (both from Roche). The cell suspension was passed through nylon filter (40 μm) and then processed for cell sorting and RNA extraction or directly for RNA extraction or protein analysis. RNA was transcribed into cDNA, and analysed as described before^66^. Briefly, random primers and MMLV Reverse Transcriptase (Invitrogen) were used for cDNA synthesis. Transcripts were quantified by real-time quantitative PCR on an ABI PRISM 7,700 Sequence Detector (Perkin-Elmer Applied Biosystems) with Applied Biosystems predesigned TaqMan Gene Expression Assays and reagents according to the manufacturer's instructions. The following probes were used (identified by Applied Biosystems assay identification number): *Tbx21* (Mm00450960_m1), *Rorc* (Mm01261019_m1), *Ahr* (Mm00478932_m1), *Il23r* (Mm00519943_m1), *Il1b* (Mm01336189_m1), *Il-18* (Mm00434226_m1), *Il23* (Mm00518984_m1), *Il1a* (Mm00439620_m1), *Il6* (Mm00446190_m1). For each sample, mRNA abundance was normalized to the amount of 18S rRNA and expressed as arbitrary units.

### Proteome profiler

Lymph node cells were lysed using RIPA lysis buffer and proteinase inhibitors (Roche) to extract proteins. Protein concentration in the tissue lysates was determined by bicinchoninic acid Protein Assay Reagent (Pierce). Proteins from tissue lysates (70 μg) were analysed using a commercial kit (Mouse cytokine array panel A Array kit, R&D Systems) containing nitrocellulose membranes coated with 40 different anti-cytokine or anti-chemokine antibodies printed in duplicate, following the manufacturer's instructions. Chemiluminescenc was measured by ImageQuantum LAS4000 (GE Healthcare Life Science) and signals quantified using AIDA Evaluation Software (BioImaging).

### ELISA

To measure cytokine concentration in cell culture supernatants, the following ELISA kits were used: IL-1β (R&D Systems), IFN-γ (BD Biosciences), IL-17A (BD Biosciences), IL-22 (Antigenix America), GM-CSF (eBioscience), IL-10 (eBioscience), IL-6 (R&D System). ELISA assays were performed according to the manufacturer's instructions.

### Statistics

Data were analysed with Prism 5 (GraphPad Software) using the nonparametric unpaired Mann–Whitney *U*-test. Graphs show the mean+standard error of the mean (s.e.m.). *P<0.05, **P<0.01, ***P<0.001, ****P<0.0001.

## Additional information

**How to cite this article:** Ronchi, F. *et al.* Experimental priming of encephalitogenic Th1/Th17 cells requires pertussis toxin-driven IL-1β production by myeloid cells. *Nat. Commun.* 7:11541 doi: 10.1038/ncomms11541 (2016).

## Supplementary Material

Supplementary Information Supplementary Figures 1-12
