# Supplementary material for: Experimental priming of encephalitogenic Th1/Th17 cells requires pertussis toxin-driven IL-1β production by myeloid cells
Source: Nat Commun. 2016 May 18;7:11541. doi: 10.1038/ncomms11541 (PMC4873938; doi:10.1038/ncomms11541)
Supplement: Supplementary Information — Supplementary Figures 1-12 [file ncomms11541-s1.pdf]

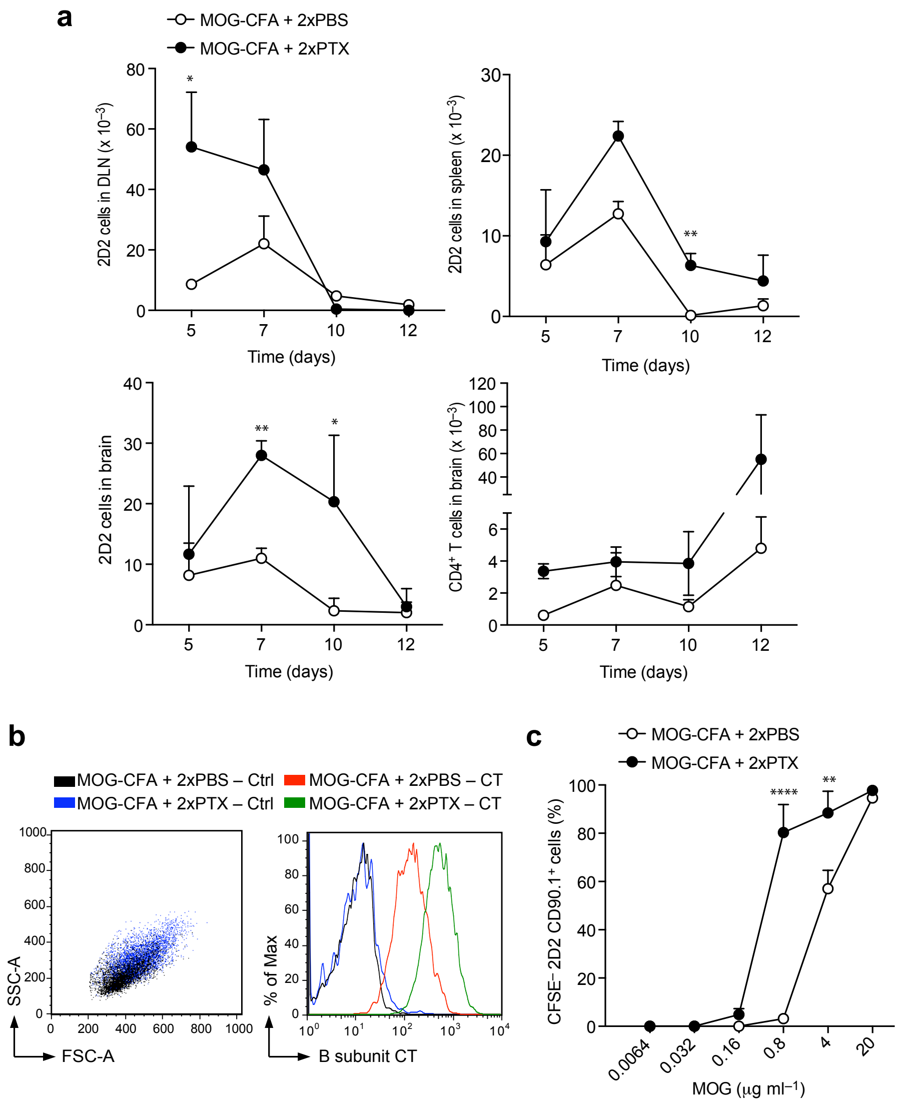

Supplementary Figure 1: PTX synergizes with CFA to promote expansion of highly responsive 2D2 T cells.

(a) Absolute number of $CD90.1^+$ $CD4^+$ TCR-transgenic 2D2 T cells in DLN, spleen and brain and of endogenous $CD90.1^-$ $CD4^+$ T cells in brain of wt mice, which had been adoptively transferred with naïve 2D2 T cells, at different time points after immunization with MOG-CFA and injection of PBS or PTX on day 0 and day 2. Data are mean + s.e.m. (n=3) and are representative of more than five independent experiments with at least three mice per group. **(b)** Forward and side scatter (left) and staining with FITC-labelled cholera toxin (CT) B subunit (right) of 2D2 T cells from DLN of wt mice on day 5 after immunization. Data are representative of three independent experiments with at least three mice per group. **(c)** Percentage of $CFSE^-$ 2D2 T cells proliferating *in vitro* to MOG_{35-55}. $CD90.1^+$ $CD4^+$ 2D2 T cells were isolated from DLN of immunized mice and stimulated *in*

vitro with bone marrow-derived LPS-matured DCs pulsed with the indicated doses of MOG_{35-55}. Proliferation, as measured by CFSE dilution, was assessed after 72 hrs of culture. Data are mean + s.e.m. (n=3) and are representative of three independent experiments with at least three mice per group. *, $P < 0.05$; **, $P < 0.01$; ****, $P < 0.0001$, as determined by nonparametric unpaired Mann-Whitney test. When not indicated, differences were not statistically significant.

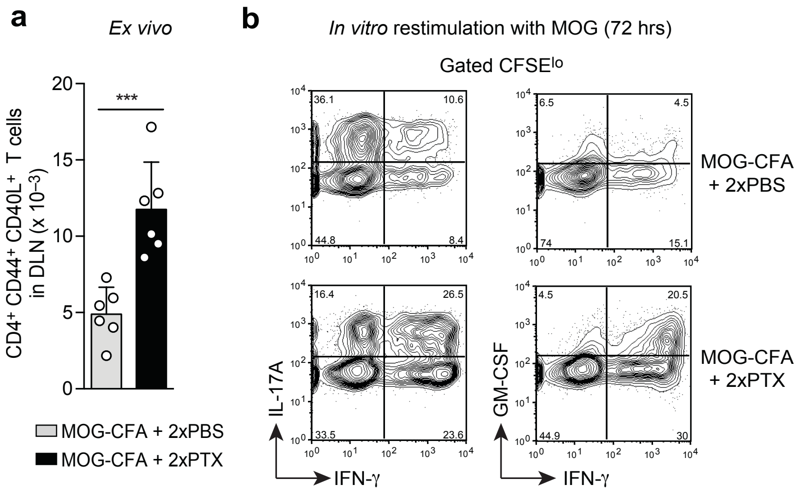

Supplementary Figure 2: PTX induces encephalitogenic Th1/17 cells.

(a) Absolute number of $CD4^+$ $CD44^+$ $CD40L^+$ endogenous T cells measured on day 5 in DLN of wt mice (with no adoptive transfer of 2D2 T cells) after immunization with MOG-CFA and injection of PBS or PTX on day 0 and day 2. Each symbol represents an individual mouse (n=6). Data are representative of more than three independent experiments, with at least three mice per group. **(b)** Representative flow cytometry analysis of intracellular cytokine staining of proliferating MOG-reactive $CFSE^-$ $CD4^+$ T cells. $CD4^+$ $CD44^+$ T cells were isolated from DLN of immunized mice, labelled with CFSE and stimulated *in vitro* for 72 hrs with $MOG_{35\text{-}55}$-pulsed splenocytes. T cells were then restimulated for 5 hrs with PMA and ionomycin in the presence of BFA for the last 3 hrs before intracellular staining. Data are representative of more than three independent experiments with at least three mice per group. Numbers represent percentage of cells in each quadrant. $P < 0.001$, *** as determined by nonparametric unpaired Mann-Whitney test.

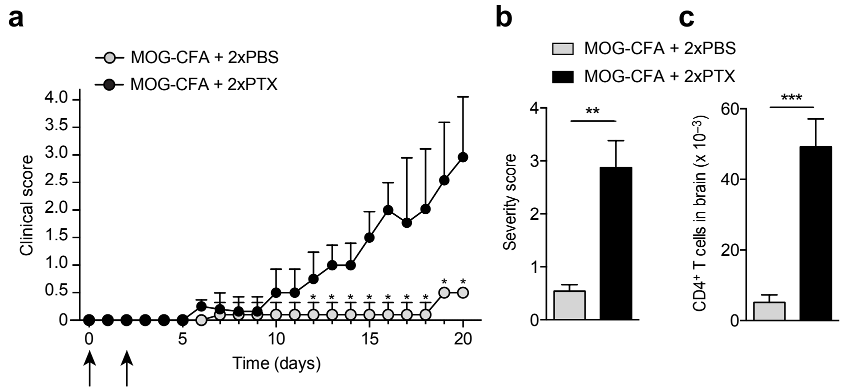

Supplementary Figure 3: PTX increases the incidence and severity of EAE.

(a) Clinical scores of C57BL/6 mice at various times after immunization with MOG-CFA and injection of PBS or PTX on day 0 and day 2 (arrows). Data are mean + s.e.m. (n=5) and are representative of more than 5 independent experiments with 4-5 mice per group. (**b**) Severity score of mice shown in a (day 20). Data are mean + s.e.m. (n=7) and are representative of more than 5 independent experiments with 4-5 mice per group. **(c)** Absolute number of endogenous $CD4^+$ T cells in brain and spinal cord of wt mice on day 5 after immunization. Data are mean + s.e.m. (n=5) and are representative of more than three independent experiments with 5-7 mice per group. *, $P < 0.05$, **, $P < 0.01$, ***, $P < 0.001$, as determined by nonparametric unpaired Mann-Whitney test.

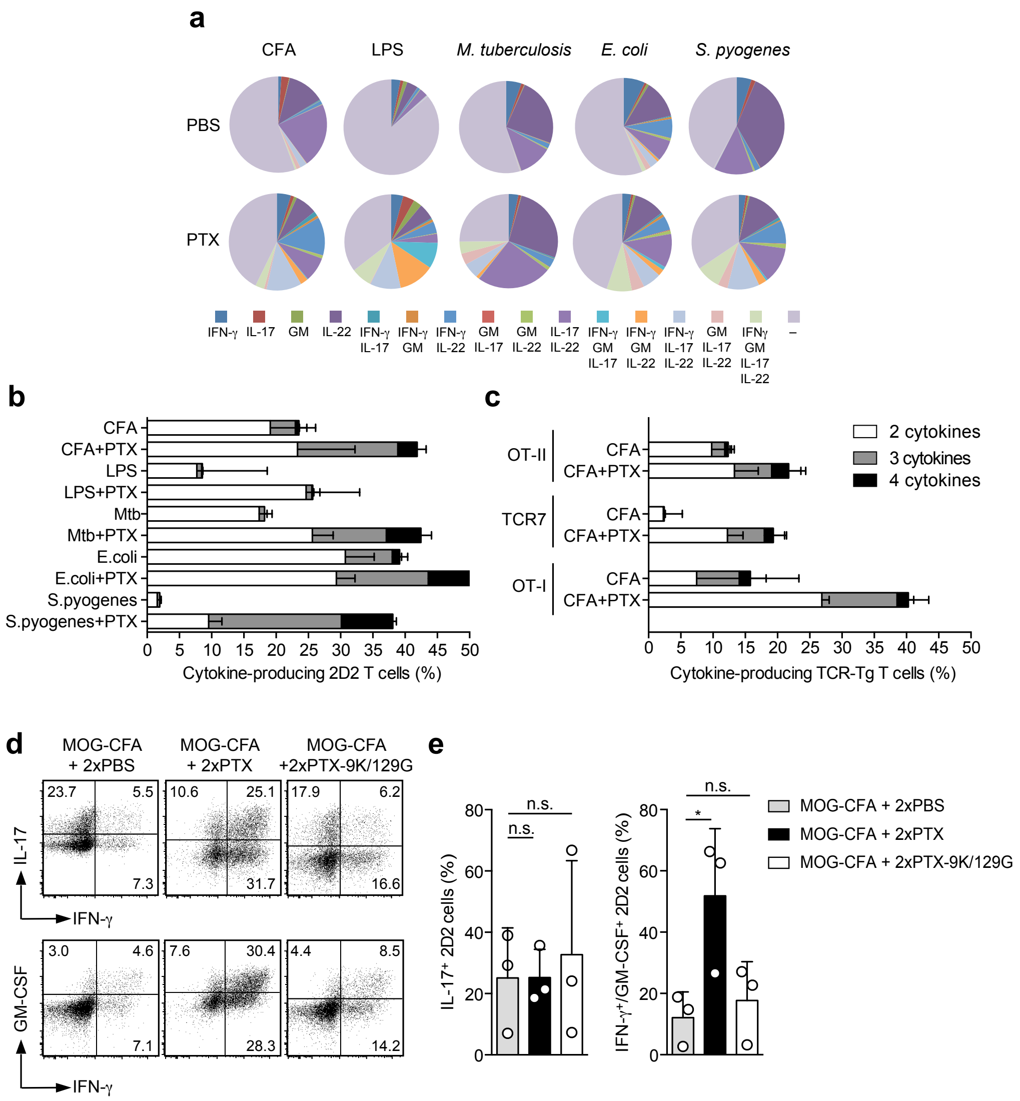

Supplementary Figure 4: Enzymatically active PTX synergizes with several TLR agonists for priming of multifunctional T cells.

(a) Boolean gating analysis showing each possible combination of production of IFN-γ, IL-17A, IL-22 and GM-CSF by 2D2 T cells in DLN of mice immunized with MOG together with either CFA, LPS, *M. tuberculosis*, *E. coli*, or *S. pyogenes* and injected with PBS or PTX on day 0 and day 2. **(b)** Histogram of the data shown in a. Percentage of 2D2 T cells producing 2, 3 or 4 cytokines (IFN-γ, IL-17A, IL-22 and GM-CSF) in different combinations in DLN of immunized mice. Data are mean+ s.e.m. (n=3) are representative of 3 independent experiments with 3 mice per group. **(c)** Percentage of OT-II, TCR7, or OT-I transgenic T cells producing 2, 3 or 4 cytokines (IFN-γ, IL-17A, IL-22 and GM-CSF) in different combinations in DLN of mice immunized with the relevant antigen in CFA.

Data are mean+ s.e.m. (n=3) are representative of 2 independent experiments with 3 mice per group. (**d**) Representative flow cytometry analysis of cytokine staining of 2D2 T cells producing IL-17, GM-CSF and IFN-γ in DLN on day 5 after immunization with MOG-CFA and injection of PBS, PTX, or enzymatically inactive PTX-9K/129G on day 0 and 2. For intracellular cytokine staining, cells were stimulated *ex vivo* for 5 hrs with PMA and ionomycin, in the presence of BFA for the last 3 hrs. (**e**) Percentage of 2D2 T cells producing IL-17, GM-CSF and/or IFN-γ in DLN on day 5 after immunization. Data are mean + s.e.m. (n=3) and are representative of three independent experiments with at least three mice per group. Each symbol represents an individual mouse. *, $P < 0.05$, as determined by nonparametric unpaired Mann-Whitney test.

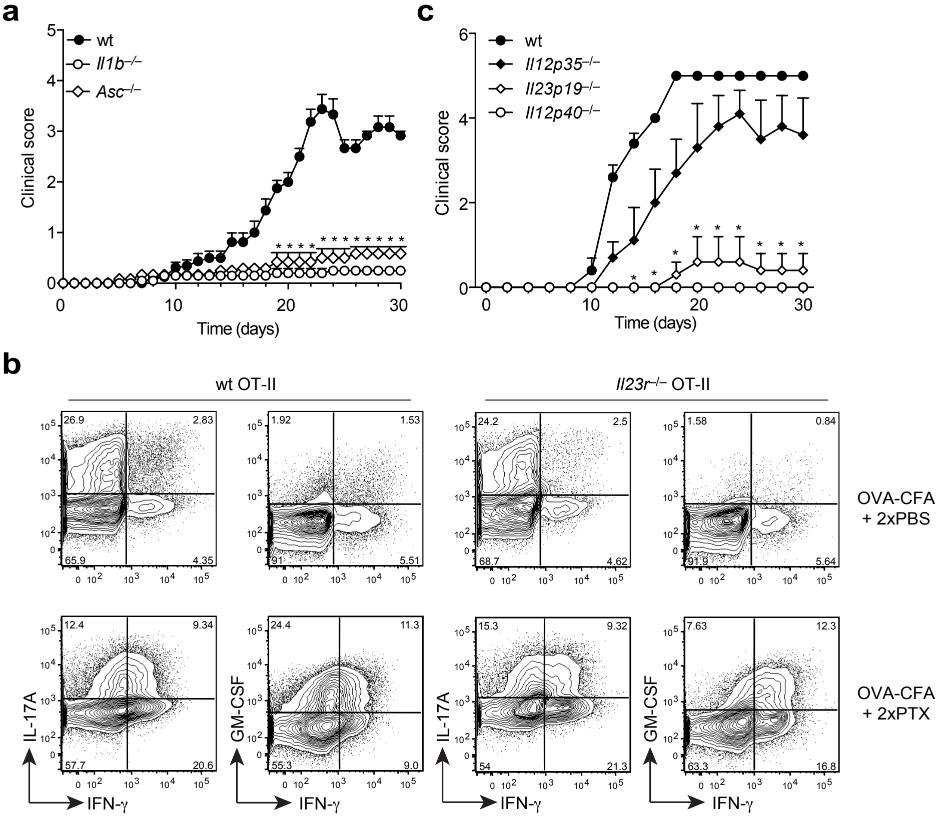

Supplementary Figure 5: ASC-, IL-1β- and IL-23-deficient mice are resistant to EAE. **(a)** Clinical scores of wt, *Asc*$^{-/-}$ and *Il1b*$^{-/-}$ mice at various times after immunization with MOG-CFA and injection of PTX on day 0 and day 2. Data are mean + s.e.m. (n=5-7) and are representative of more than three independent experiments with at least five mice per group. **(b)** Representative flow cytometry analysis of cytokine staining of wt or *Il23r*$^{-/-}$ OT-II cells producing IL-17, GM-CSF and IFN-γ in DLN on day 5 after immunization with OVA-CFA and injection of PBS or PTX on day 0 and day 2. For intracellular cytokine staining, cells were stimulated *ex vivo* for 5 hrs with PMA and ionomycin, in the presence of BFA for the last 3 hrs. Data are from pool of six lymph nodes/group from three immunized mice and are representative of two independent experiments. **(c)** Clinical scores of wt, *Il12p35*$^{-/-}$, *Il12p40*$^{-/-}$, and *Il23p19*$^{-/-}$ mice at various times after immunization with MOG-CFA and injection of PTX on day 0 and 2. Data are mean + s.e.m. (n=5) and are representative of more than three independent experiments with at least five mice per group. *, $P < 0.05$, as determined by nonparametric unpaired Mann-Whitney test.

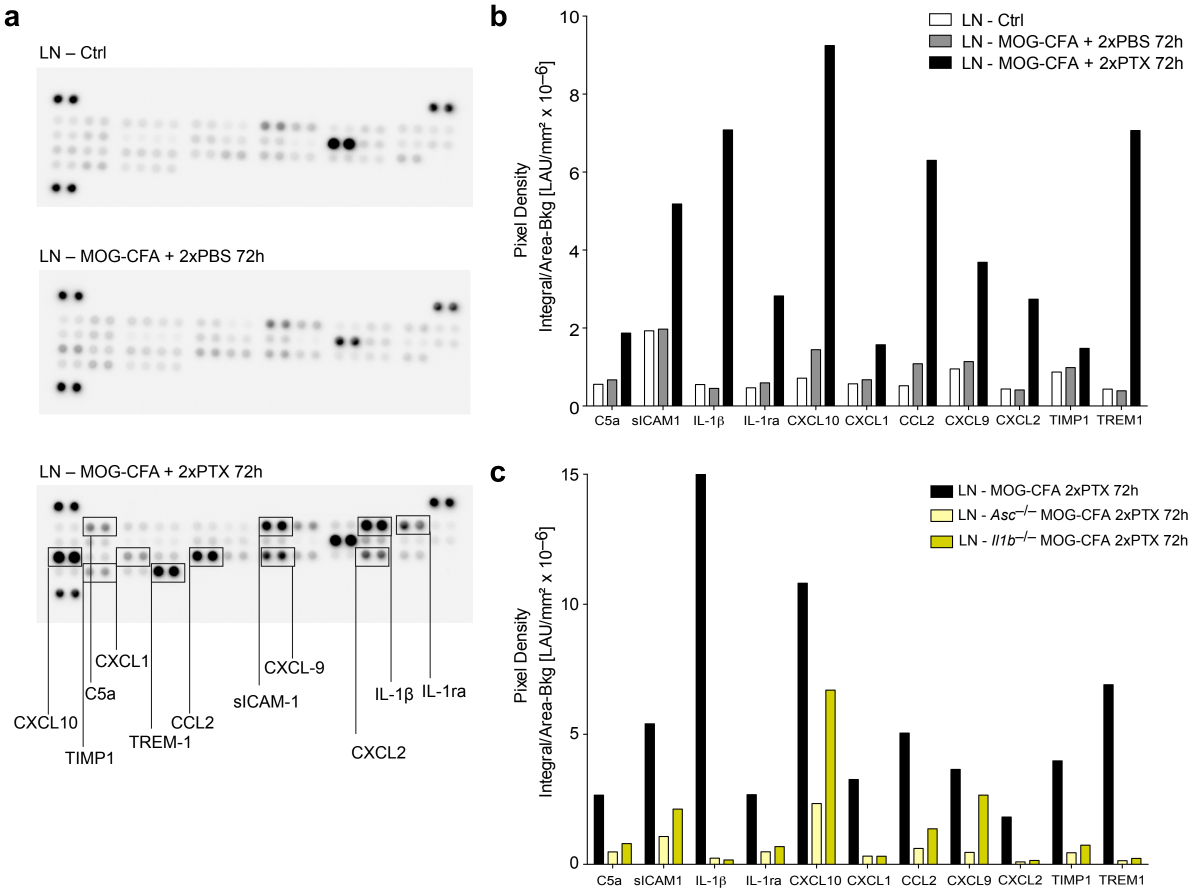

Supplementary Figure 6: (a) Proteome profiler assay on lymph node lysates (LN) from wt mice not immunized (Ctrl) or 72 hrs after immunization with CFA and injection of PBS or PTX on day 0 and day 2. Organs were lysed and proteins were extracted and loaded onto nitrocellulose membranes. Protein samples were from a pool of six lymph nodes/group from three immunized mice. Data are representative of three independent experiments. **(b)** Quantification of the cytokine array data shown in a. **(c)** Quantification of the cytokine array data performed on lymph node lysates (LN) from wt, *Asc*$^{-/-}$ and *Il1b*$^{-/-}$mice 72 hrs after immunization with CFA and injection of PTX on day 0 and day 2. Data are from pool of six lymph nodes/group from three immunized mice. A modest increase of IL-1β, CXCL1 and CXCL2 was detected in the blood 6 hrs after the second injection of PTX.

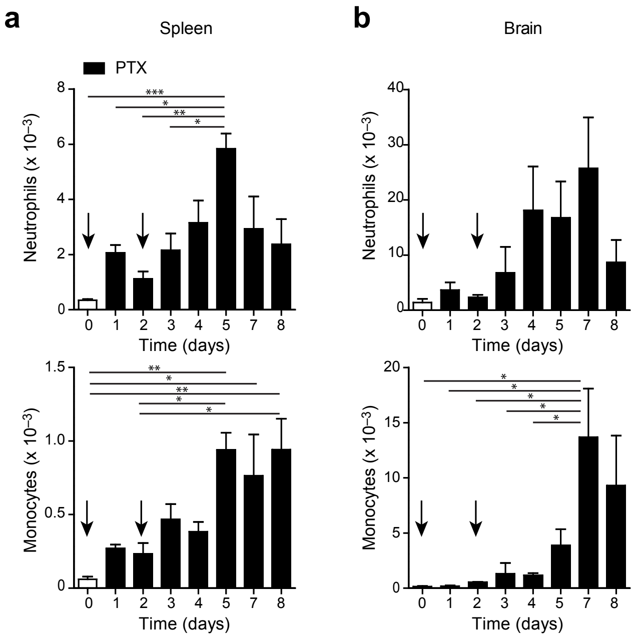

Supplementary Figure 7: Injection of PTX alone induces inflammatory monocyte and neutrophil recruitment in the spleen and brain of wt mice.

(a, b) Absolute number of $CD11b^{+}$ $Gr1^{hi}$ $Ly6C^{int}$ $Ly6G^{+}$ neutrophils or $CD11b^{+}$ $Gr1^{int}$ $Ly6C^{hi}$ $Ly6G^{-}$ monocytes in spleen and brain at different time points following injection of PTX alone on day 0 and on day 2 (as indicated by the arrows). Data are mean + s.e.m. and are representative of more than three independent experiments with at least three mice per group. *, $P < 0.05$; **, $P < 0.01$; ***, $P < 0.001$, as determined by nonparametric unpaired Mann-Whitney test.

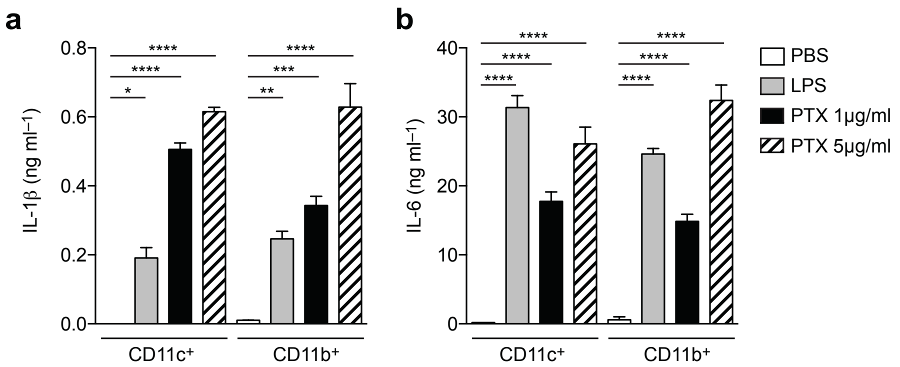

Supplementary Figure 8: PTX induces production of IL-1β by CD11c$^+$ and CD11b$^+$ myeloid cells *in vitro*. Production of IL-1β **(a)** and IL-6 **(b)** by CD11c$^+$ and CD11b$^+$ myeloid cells sorted by MACS from spleen and lymph nodes of wt mice and cultured *in vitro* (10^5 cells/well in 96-well plate) for 16 hrs in the presence of the indicated stimuli. ATP was added for the last 45 min of culture. Data are mean + s.e.m. (n=3) and are representative of two independent experiments. *, $P < 0.05$; **, $P < 0.01$; ***, $P < 0.001$; ****, $P < 0.0001$, as determined by nonparametric unpaired Mann-Whitney test.

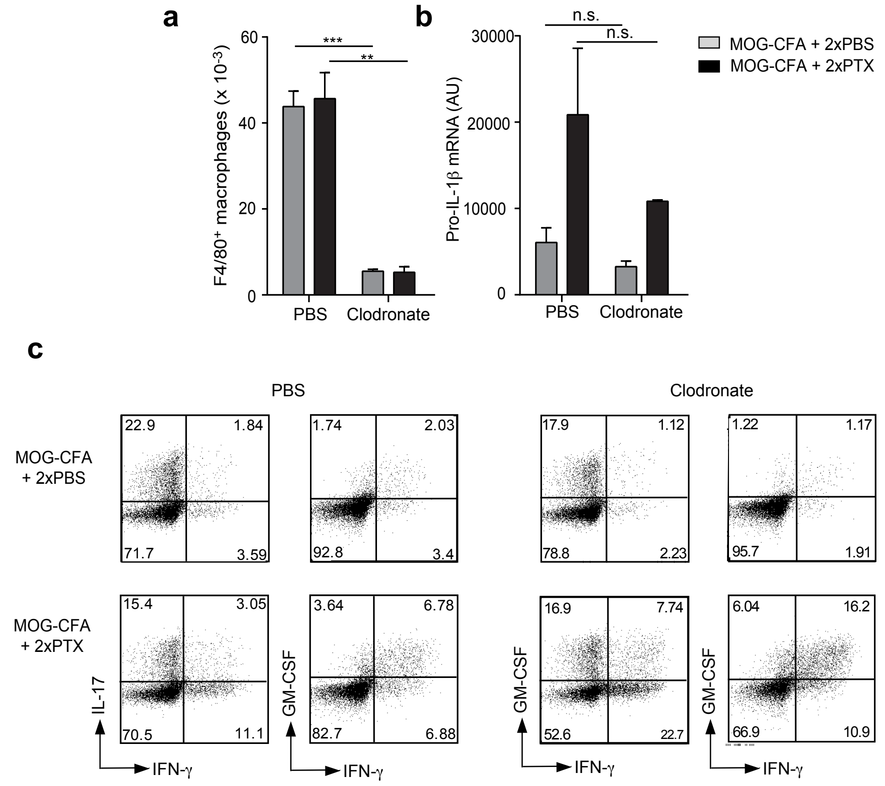

Supplementary Figure 9: PTX effect in macrophage depleted mice

(**a,b**) Absolute numbers of $CD11b^{+}$ $F4/80^{+}$ $Gr1^{low}$ cells and pro-IL-1β mRNA abundance 72 hrs after immunization in DLN of mice that had been treated with clodronate-containing liposomes (clodronate) or PBS liposomes (PBS). Data are mean + s.e.m. (n=3) and are representative of two independent experiments.**, $P < 0.01$; ***, $P < 0.001$, as determined by nonparametric unpaired Mann-Whitney test. (**c**) Representative flow cytometry analysis of intracellular cytokine staining of 2D2 T cells from DLN of immunized clodronate- or PBS-treated mice. Cells were restimulated *in vitro* for 5 hrs with PMA and ionomycin, in the presence of BFA for the last 3 hrs. Numbers indicate percentage positive cells in each quadrant. Data are from pool of six lymph nodes/group from three immunized mice and are representative of two independent experiments.

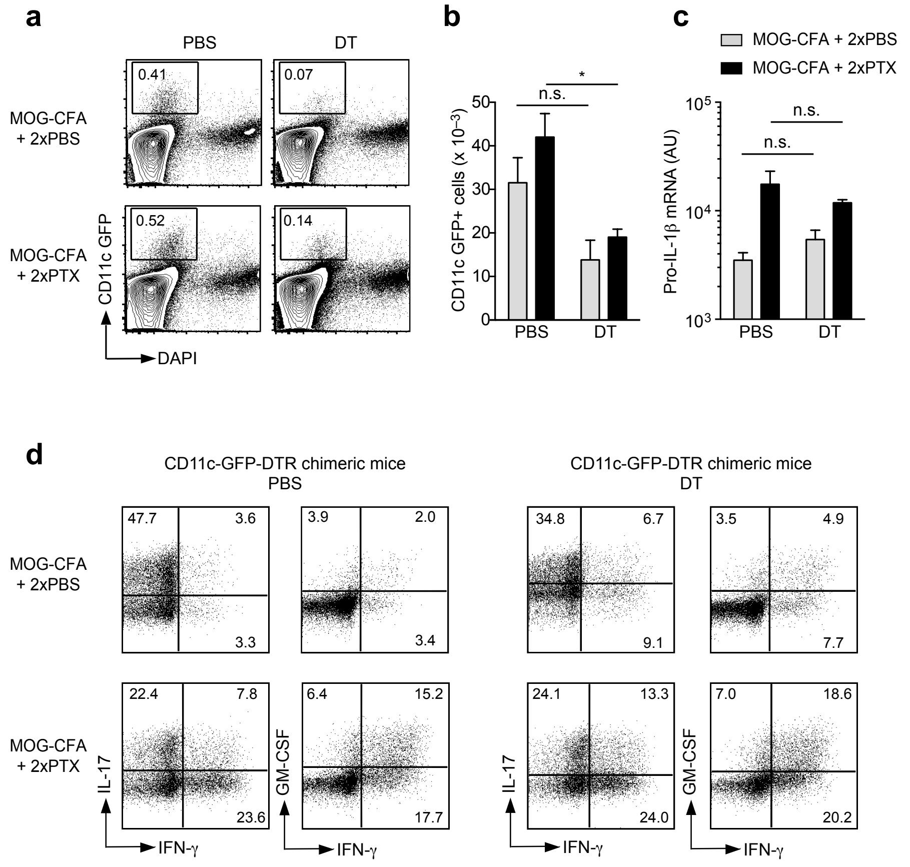

Supplementary Figure 10: PTX effect in CD11c-GFP-DTR chimeric mice

(a) Flow cytometry analysis of CD11c-GFP$^+$ in CD11c-GFP-DTR chimeric mice after i.p. administration of PBS (left panel) or DT (right panel) and immunization with MOG-CFA and injection of PBS or PTX on day 0 and day 2. (**b,c**) Absolute numbers of CD11c-GFP$^+$ cells and pro-IL-1β mRNA abundance in DLN of CD11c-GFP-DTR chimeric mice 72 hrs after immunization. Data are mean + s.e.m. (n=3) and are representative of two independent experiments. *, $P < 0.05$, as determined by nonparametric unpaired Mann-Whitney test. (**d**) Representative flow cytometry analysis of intracellular cytokine staining of 2D2 T cells from DLN of immunized CD11c-GFP-DTR chimeric mice. Cells were restimulated *in vitro* for 5 hrs with PMA and ionomycin, in the presence of BFA for the last 3 hrs. Numbers indicate percentage positive cells in each quadrant. Data are from pool of six lymph nodes/group from three immunized mice and are representative of three independent experiments.

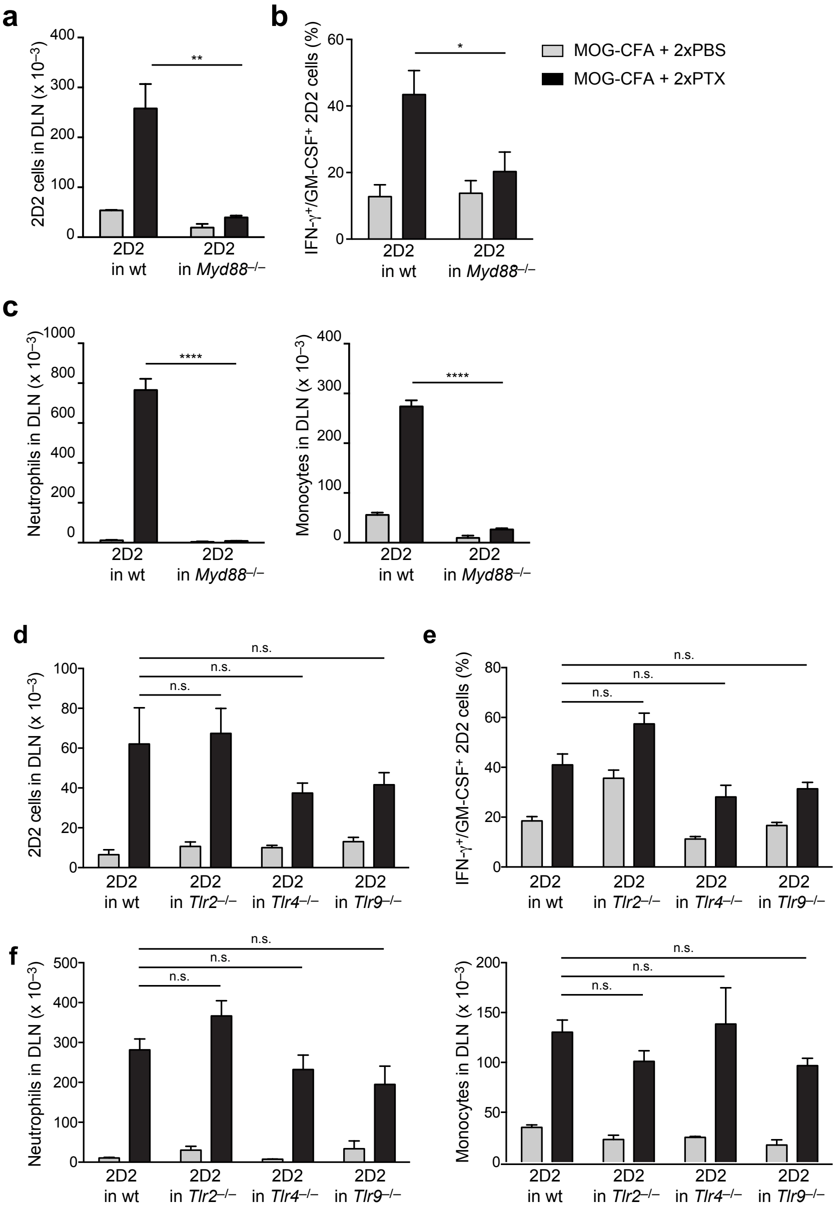

Supplementary Figure 11: PTX requires MyD88 signalling on non-T cells.

(a,b) Absolute number of CD90.1$^+$ CD4$^+$ 2D2 T cells (a) and percentage of IFN-γ$^+$/GM-CSF$^+$ 2D2 T cells (b) in DLN of wt or *Myd88*$^{-/-}$ hosts immunized 5 days earlier with MOG-CFA and injected with two doses of PBS or PTX on day 0 and day 2. **(d,e)** Absolute number of CD90.1$^+$ CD4$^+$ 2D2 T cells (d) and percentage of IFN-γ$^+$/GM-CSF$^+$ 2D2 T cells (e) in DLN of wt or *Tlr*$^{-/-}$ hosts immunized 5 days earlier with MOG-CFA and injected with two doses of PBS or PTX on day 0 and day 2. **(c,f)** Absolute number of CD11b$^+$ Gr1^{hi} Ly6C^{int} Ly6G$^+$ neutrophils and CD11b$^+$ Gr1^{int} Ly6C^{hi} Ly6G$^-$ monocytes in DLN of wt or *Myd88*$^{-/-}$ hosts (c) or wt and *Tlr*$^{-/-}$ hosts (f) on day 5 after immunization with MOG-CFA

and injection of 2 doses of PBS or PTX on day 0 and day 2. Data are from pool of six lymph nodes/group from three immunized mice and are representative of five independent experiments. *, $P < 0.05$, **, $P < 0.01$; ****, $P < 0.0001$, as determined by nonparametric unpaired Mann-Whitney test.

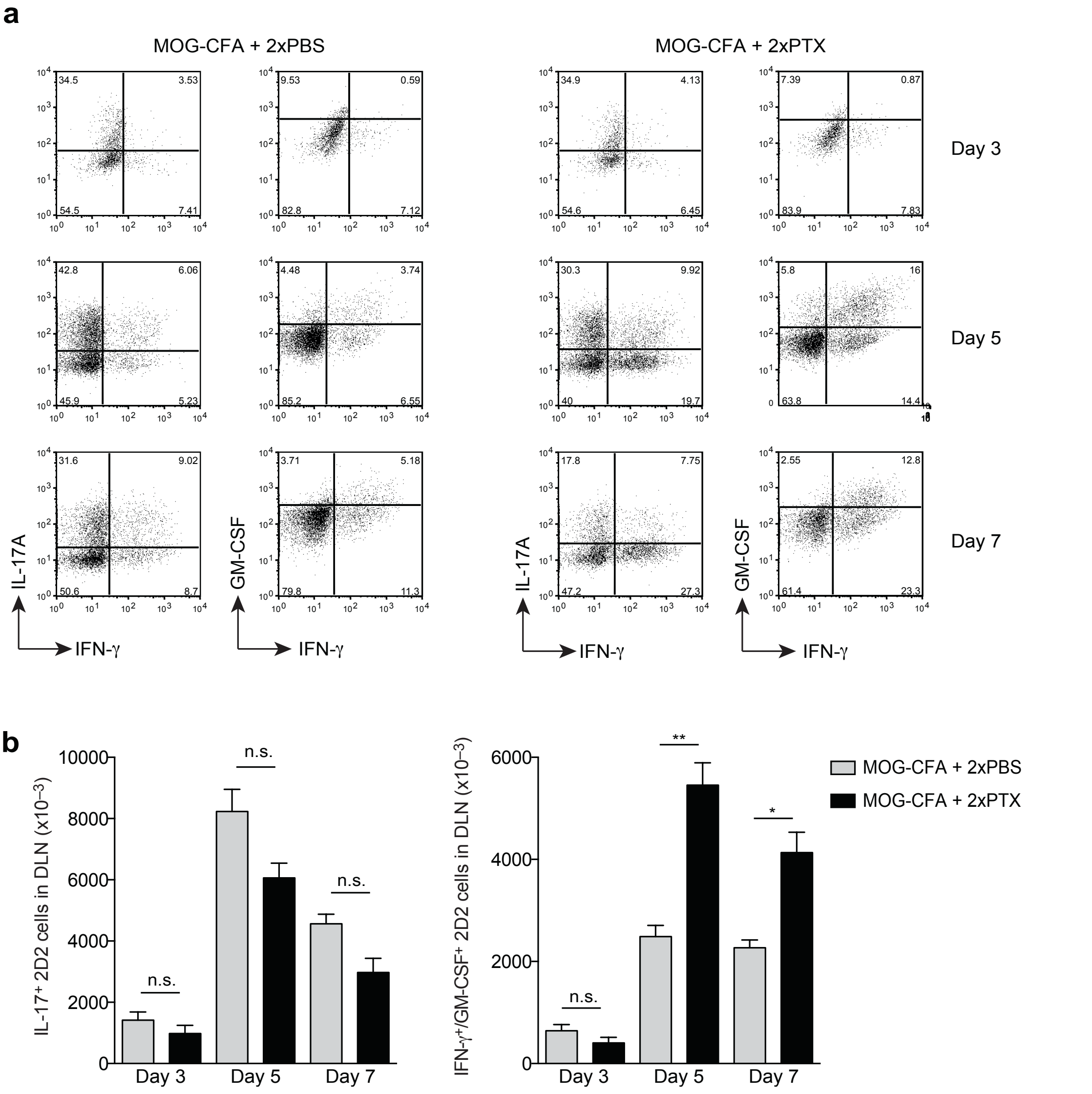

Supplementary Figure 12: Cytokine production by 2D2 T cells at different time points after immunization.

(a) Representative flow cytometry analysis of $CD90.1^+$ 2D2 T cells in DLN of mice at different time points after immunization with MOG-CFA and injection of PBS or PTX on day 0 and day 2. Cells were restimulated *in vitro* for 5 hrs with PMA and ionomycin, in the presence of BFA for the last 3 hrs. Numbers indicate percentage positive cells in each quadrant. **(b)** Absolute number of IL-17^+ and IFN-γ^+/GM-CSF^+ $CD90.1^+$ 2D2 T cells in DLN of mice at different time points after immunization with MOG-CFA and injection of PBS or PTX on day 0 and day 2. Data are mean + s.e.m. (n=3-6) from two independent experiments with at least three mice per group. *, $P < 0.05$, **, $P < 0.01$, as determined by nonparametric unpaired Mann-Whitney test.
